# Dynamics near equilibria in the Jupiter-Europa system using the Lie-series technique

**DOI:** 10.1038/s41598-026-60837-8

**Published:** 2026-07-14

**Authors:** Dina Tarek, Magdy A. Sirwah, M. Radwan, H. R. Dwidar

**Affiliations:** 1 Mathematics Dept., Faculty of Science, Tanta University, Tanta, Egypt; 2 Astronomy, Space Science and Meteorology Dept., Faculty of Science, Cairo University, 12613 Giza, Egypt

**Keywords:** Lie-series method, Libration points, Tadpole and horseshoe orbits, Astronomy and planetary science, Engineering, Mathematics and computing, Physics

## Abstract

Investigating the dynamics near planetary satellites is crucial for space mission design. This work investigates the dynamics around the equilibrium points in the Jupiter-Europa system in the restricted three-body problem framework. The current dynamical model incorporates perturbations due to the oblateness of both Jupiter and Europa, as well as the equatorial ellipticity of Europa. We constructed the equations of motion under the effects of the perturbations considered. Then, we applied the Lie-integration method to solve the equations of motion and derive an analytical approximate solution. Also, we used the C++ software package CVODE to validate our results. The results confirmed close agreement between the Lie series and the numerical approaches. Using the Lie-series method, we applied different initial conditions to generate horseshoe and tadpole orbits around the equilateral points in both the classical circular restricted three-body problem and the perturbed model. In addition, we investigated the linear stability of the Jupiter–Europa system and found that it remains stable up to a critical mass ratio of $\mu _c=0.0249882790$. Furthermore, we used the zero-velocity surfaces to analyze the accessibility condition in the Jupiter-Europa system. We found that the spacecraft transit between the primaries remains possible until the Jacobi constant exceeds the value 3.00382022.

## Introduction

The restricted three-body problem is a fundamental model in celestial mechanics. It describes the motion of an infinitesimal body under the gravitational influence of two primary bodies. The two primaries orbit their common center of mass in circular or elliptic orbits. By ignoring the particle’s effect on the two primaries, this problem provides a simplified framework for studying complex gravitational situations. Its significance lies in the ability to capture important dynamical features like equilibrium points, families of periodic orbits, stability, and surfaces of zero velocity. These features govern the accessibility and stability of trajectories in multi-body dynamical systems. In space operations, the restricted three-body problem has many applications, such as capturing the dynamics near moons and planets^1^^,^^2^.

The Jovian system comprises Jupiter, and a diverse population of natural satellites, form one of the most complex planetary systems. At least 95 known moons orbit Jupiter, ranging in size from small, irregular objects to the four Galilean moons. These planetary satellites exhibit remarkable geophysical diversity and physical characteristics. The dynamics of the Jupiter-Europa system are governed by the powerful gravitational pull of Jupiter and the orbital resonances between its largest moons. Europa holds significant scientific interest because tidal forces from Jupiter and its resonance with the Galilean moons generate internal heating. Europa’s shape deviates slightly from a perfect sphere, driven by the joint effects of Jupiter’s tidal forces, its rotation, and internal structure. Artificial satellites orbiting Europa are subject to a complex dynamical environment. This complexity is mainly due to Jupiter’s gravity, Europa’s deviation from a spherical shape, and perturbations from neighboring moons. The complex dynamical environment causes perturbations in satellite orbits, leading to variations in orbital elements over time^3^^,^^4^^,^^5^.

Spacecraft operating in the vicinity of Europa can be modeled as a small particle, subject to the combined gravitational field of Jupiter and Europa. This assumption reduces the complexity of the equations of motion while capturing the dynamical characteristics of the system. To go beyond the classical restricted three-body problem, authors need to use both qualitative and quantitative mathematical tools that capture the dynamical features of the system. Qualitative analysis, such as Poincaré maps and surfaces of zero velocity are used to reveal the global structure of trajectories, the onset of chaos, etc. These tools allow researchers to identify dynamical highways for low-energy transfers and classify motion near libration points. Quantitatively, modern approaches utilize normal form theory and perturbation methods to approximate periodic solutions and assess their stability. Numerical techniques, such as multiple shooting algorithms, provide robust computational frameworks for long-term trajectory prediction. Together, these mathematical tools enable both theoretical advances and practical applications in space operations^6^^,^^7^.

In celestial mechanics, a periodic orbit is a path along which a spacecraft, subject to a gravitational field, can return to its initial state vector after a certain period. Periodic orbits play a key role in the restricted three-body problem for several reasons. Determining a periodic orbit reveals a recurrent pattern of motion, making predictions about future state vectors of the bodies easier. These orbits can be used to classify the types of motion that occur in the restricted three-body problem. Additionally, the stability of the system can be determined by periodic orbits. In the restricted three-body system, the existence of periodic orbits is governed by the dynamic equilibrium between gravity and centrifugal forces. When these forces counterbalance one another, the probes can form a periodic motion in the vicinity of a specific location^7^.

Near the collinear points $(L_i, i=1,2,3)$, probes can form Lyapunov and halo orbits. Both types are periodic near collinear points. The first type is a two-dimensional plane motion, while the second is a three-dimensional space motion. The triangular points $L_i, i=4, 5$, are naturally stable, allowing engineers to design a variety of useful orbits around them for space mission applications. The stable oscillations about the equilateral points $L_i, i=4, 5$, are referred to as tadpole orbits. The horseshoe orbits represent a class of solutions to the circular restricted three-body problem, which arises when investigating the Lagrange points. Understanding dynamics near the triangular points is facilitated by studying the horseshoe and tadpole orbits^8^. Periodic orbits can be classified into stable and unstable periodic orbits, depending on their motion after perturbations. Stable orbits (such as $L_i, i=4,5$) return to their original state after being subjected to minor perturbations. This makes them suitable for long-period stationing missions. Unstable orbits, such as the $L_2$ halo orbit, require regular maintenance to remain stable. However, their advantage is the ability to perform orbit transfer with little thrust. Furthermore, analyzing the zero velocity surfaces around the points defines the energetic thresholds for orbit families and serves as an essential tool for evaluating long-term stability^9^^,^^10^^,^^11^^,^^12^^,^^13^^,^^14^.

Tadpole orbits in the triangular Lagrange regions $L_4/ L_5$ can be used in space missions to transfer probes to a horseshoe orbit via the stable manifolds connecting the two types of orbits. By leveraging these low-energy trajectories, mission design can achieve efficient transport with reduced propellant cost. Such transfers are valuable for studying libration regions and exploring Jupiter’s moons^15^. Our numerical and approximate analytical methods construct both tadpole and horseshoe orbits, advancing the understanding of libration regions and facilitating studies of dynamical behavior near Jupiter’s moons. The work accounts for small perturbations, which are crucial for planetary defense, navigation, and extended-duration missions. The accessibility conditions further enable engineers to exploit natural dynamical pathways for fuel-efficient transfers between Jupiter and Europa, while the identification of stability regions allows mission planners to extend spacecraft lifetimes in the system.

Researchers have extensively investigated small-particle dynamics within the restricted three-body problem framework, employing various approaches and techniques to account for perturbations. Rape^16^, found two sets of initial conditions for horseshoe and for long tadpole-shaped orbits around the equilateral points. Taylor^17^, described the smooth horseshoe orbits around the equilateral points with different initial conditions in the Sun-Jupiter system. Howell and Campbell^18^, used $L_1$ and $L_2$halo orbits as baseline families and identified many bifurcations and intersections representing the existence of other three-dimensional families. Hamdy et al.^19^, derived explicit analytical expressions for the small-amplitude motion of an infinitesimal mass around the equilibrium points in the elliptic restricted three-body problem. Using the Lie series method, they performed an analytical integration of the equations of motion and obtained solutions to the canonical system. In addition, Yárnoz et al.^20^, explored families of planar symmetric periodic orbits around minor bodies under solar radiation pressure perturbation. Recently, Ferreira and Prado^21^, analyzed periodic orbits in the restricted three-body problem that pass close to the smaller primary. Ibrahim et al.^14^,studied the dynamical properties of Hill’s system under perturbation due to the continued fraction. Furthermore, they defined the zero-velocity curves and the regions of possible motion at different Jacobi constants.

Scientific questions motivate new space mission proposals from different space agencies. The planetary satellites of Jupiter, Saturn and Uranus may host habitable environments. Furthermore, in situ observation may provide new insights into the understanding of the solar system’s formation and evolution. In the current work, we use the model proposed by Chen et al.^22^to study the Jupiter-Europa system from other aspects. In Yousuf et al.^23^, the authors analyzed the equilibrium points and associated periodic orbits considering the oblateness of both Jupiter and Europa. We extend their work and consider the equatorial ellipticity of Europa. We use the Lie series technique to obtain an approximate analytical solution to the system’s equation of motion. Then, we use this analytical solution to generate the tadpole and horseshoe orbits in the neighborhood of triangular points. In addition, We examine the linear stability of the Jupiter–Europa system under the influence of the included perturbations. Furthermore, We use the surfaces of zero velocity method to examine the accessibility conditions.

## Dynamical model and equations of motion

Here, we investigate the planar version of the Jupiter-Europa system in the restricted three-body problem framework. Jupiter and Europa are treated as the two primaries of the system. The two bodies rotate about their center of mass in a circular orbit under their mutual gravitational pull. Additionally, a small third body (spacecraft) moves within the plane under the gravitational field of both bodies. Initially, both Jupiter and Europa are aligned along the x-axis, where Europa is located on the positive side. Unlike the classical restricted problem, Jupiter’s oblateness and the non-spherical gravity field of Europa are incorporated. For convenience, the system is expressed using dimensionless variables. Let $m_1$ and $m_2$ be the masses of both Jupiter and Europa, respectively. Assuming that the sum of masses of the primaries is the unit of mass, and that the distance between them is unity. Then the unit of time is such that $G(m_1+m_2)=1$.

The equations of motion of the system are expressed in a rotating frame. The origin of this synodic frame is set at the center of mass of the three-body problem. Since Europa is tidally locked to Jupiter and rotates synchronously, its body-fixed frame rotates at the same rate as the synodic-rotating frame of the circular restricted three-body problem. The synchronous rotation condition preserves the autonomous form of the dynamical model. Let the coordinates of Jupiter and Europa be $(-\mu , 0)$ and $(1-\mu , 0)$, respectively, and (*x*, *y*) be the coordinate of the third body in the synodic frame. Then, the equations of motion can be written as^22^^,^^23^.

1 $$\begin{aligned} x'' - 2y'=\frac{\partial U}{\partial x}\ , \nonumber \\ y'' + 2x'=\frac{\partial U}{\partial y}\ , \end{aligned}$$

The pseudo-potential function *U* associated with the problem is defined as^22^

2 $$\begin{aligned} U(x,y)=\left[ \frac{1}{2}\left( x^2+y^2\right) \right.&+\frac{1}{n^2}\left\{ \frac{(1-\mu )}{r_1}+\frac{(1-\mu ) J_{2,J}R^{2}_{J}}{2 r_1^3}+\frac{\mu }{r_{2}}\right. \nonumber \\&\left. \left. +\frac{\mu J_{2,E}R^{2}_{E}}{2 r_2^3}+\frac{3\mu C_{22,E}R^{2}_{E}((x-1+\mu )^2-y^2)}{ r_2^5}\right\} \right] . \end{aligned}$$

Where $R_{J}$, $R_E$, $J_{2,J}$, $J_{2,E}$, $\mu$ and $C_{22,E}$ denote, respectively, the mean radii of Jupiter and Europa, the second zonal oblateness term of Jupiter and Europa, the mass ratio of the system $\mu =\frac{m_2}{m_1+m_2}$, and the equatorial ellipticity term of Europa. The values adopted for the zonal and tesseral harmonics are presented as follows: $J_{2J} = 1.4696429006 \times 10^{-2}$, $J_{2E} = 1.9211145442 \times 10^{-4}$, and $C_{22E} = 2.0000741348 \times 10^{-4}$^12^. The mean motion, *n*, is given as

3 $$\begin{aligned} n^2&=\left[ 1+\frac{3}{2} J_{2,J}R^{2}_{J}+\frac{3}{2} J_{2,E}R^{2}_{E}\right] , \nonumber \\ r_{1}^2&=\left( x-x_1\right) ^2+y^2 \nonumber \\ r_{2}^2&=\left( x-x_{2}\right) ^2+y^2 \end{aligned}$$

where $r_1$, $r_2$ represent the distances of the spacecraft from Jupiter and Europa, respectively, while $x_1=-\mu$ and $x_{2}=1-\mu$.

## Methodology

The Lie operator provides a systematic methodology for approximating solutions for ordinary and partial differential equations. It is defined as a linear differential operator associated with a vector field. By truncating the series expansion at a finite order, this approach offers a systematic approximation for linear and nonlinear systems^6^. This technique can be outlined as follows:

4 $$\begin{aligned} D=\sum \limits _{k=0}^{N}\phi _{k}\ \frac{\partial }{\partial x_{k}} \end{aligned}$$

where $\textbf{x}=(x_{1},\ldots ,x_{N})$ lies in n-dimensional x-space. Also within a certain domain, $\phi _{i}(x)$ are holomorphic functions. By employing the operator and its power to holomorphic function *g*(*x*) in the same domain as for $\phi _{i}(x)$, we get

5 $$\begin{aligned} D g=\sum \limits _{k=0}^{N}\phi _{k}\ \frac{\partial g}{\partial x_{k}}\ \ \text {, }\ \ \ \ \ \ \ D^{n}g =D(D^{n-1}g) \end{aligned}$$

The Lie series is defined by the following expression:

6 $$\begin{aligned} L(\textbf{x},t)\ =\sum \limits _{i =0}^{\infty }\frac{t^{i }}{i !}D^{i }g(\textbf{x}),\ \ \ \ \ \ i \in \mathbb {N} \end{aligned}$$

This series converges within the domain of the function *g*, and by using the exponential function’s expansion, we can express it as:

7 $$\begin{aligned} L(\textbf{x},t)\ =e^{tD}g(\textbf{x}). \end{aligned}$$

This formulation can be applied to solve a system of ordinary differential equations (ODEs), as in previous studies in^24^^,^^25^^,^^26^, such as:

8 $$\begin{aligned} \overset{\cdot }{x}_{k}=\phi _{k}(\textbf{x}). \end{aligned}$$

The solution is given by $x_{k}=e^{tD} x_{0k}$, where $x_{0k}$ are the initial conditions, and the operator *D* is defined as $D=\sum \limits _{k=1}^{N}\phi _{k}(\textbf{x}_0)\frac{\partial }{\partial x_{0k}}$. In a similar way, the approximate solution to the equation $\dot{x}=\phi (x)$ at time $t+\Delta t$ is:

9 $$\begin{aligned} x(t+\Delta t)=L(x,\Delta t)\ =e^{\Delta tD}\ x\ (t) \end{aligned}$$

Subsequently, We will apply this method to obtain an analytical solution to the equations of motion of the current system.

## Analytical solution via Lie series technique

To obtain an approximate solution of eq. (1) using the Lie method, we first translate the coordinates of the system near any Lagrangian point using $\xi =x-\grave{x}_0$, $\eta = y-\grave{y}_0$. Where $(\grave{x}_0$, $\grave{y}_0)$ is the coordinates of the Lagrangian point. Using this Transformation, the system is converted to a first-order differential equation ($z_i, i=1\dots 4$).

Then we have

10 $$\begin{aligned} \dot{z}_1 = \dot{\xi } =\dot{x} =&z_2 \nonumber \\ \dot{z}_2 =\ddot{\xi } =\ddot{x} =&2z_4 + \left[ z_1+\grave{x}_0- \frac{1}{n^2}\left\{ \frac{(1-\mu )(z_1+\grave{x}_0+\mu )}{r_1^3}\right. \right. \nonumber \\&\left. \left. +\frac{3J_{2,J}R^{2}_{J}(1-\mu )(z_1+\grave{x}_0+\mu )}{2 r_1^5} +\frac{\mu (z_1+\grave{x}_0+\mu -1)}{r_2^3}\right. \right. \nonumber \\&+\frac{3\mu R^{2}_{E}(z_1+\grave{x}_0+\mu -1)}{2 r_2^5}(J_{2,E}-14C_{22,E}) \nonumber \\&\left. \left. +\frac{30\mu C_{22,E}R^{2}_{E}(z_1+\grave{x}_0+\mu -1)^3}{r_2^7}\right\} \right] \nonumber \\ \dot{z}_3 = \dot{\eta } = \dot{y} =&z_4 \nonumber \\ \dot{z}_4 = \ddot{\eta } = \ddot{y} =&-2z_2 + \left[ z_3+\grave{y}_0- \frac{1}{n^2}\left\{ \frac{(1-\mu )(z_3+\grave{y}_0)}{r_1^3}\right. \right. \nonumber \\&\left. \left. +\frac{3J_{2,J}R^{2}_{J}(1-\mu )(z_3+\grave{y}_0)}{2 r_1^5}+\frac{\mu (z_3+\grave{y}_0)}{r_2^3}\right. \right. \nonumber \\&+\frac{3\mu R^{2}_{E}(z_3+\grave{y}_0)}{2 r_2^5}(J_{2,E}+14C_{22,E}) \nonumber \\&\left. \left. -\frac{30\mu C_{22,E}R^{2}_{E}(z_3+\grave{y}_0)^3}{r_2^7}\right\} \right] \end{aligned}$$

where

11 $$\begin{aligned} r_{1}^2&=\left( z_1+\grave{x}_0+\mu \right) ^2+\left( z_3+\grave{y}_0\right) ^2 \nonumber \\ r_{2}^2&=\left( z_1+\grave{x}_0+\mu -1\right) ^2+\left( z_3+\grave{y}_0\right) ^2 \end{aligned}$$

To facilitate the procedures and ensure the proper application of the technquie to the system of equations, let us define the following quantities

12 $$\begin{aligned} \mathscr {K}_{i,j}&= z_j+\delta _j^1\grave{x}_0 +\delta _j^3\grave{y}_0 +(\delta _j^1-\delta _i^3)\mu -\delta _i^2 \end{aligned}$$

13 $$\begin{aligned} \mathfrak {L}_{i,j}^{p}&= (z_j+\delta _j^1\grave{x}_0 +\delta _j^3\grave{y}_0 +(\delta _j^1-\delta _i^3)\mu -\delta _i^2)^p \end{aligned}$$

14 $$\begin{aligned} \varrho _i^p&= ((z_1+\grave{x}_0+\mu -\delta _i^2 )^2+(z_3+\grave{y}_0)^2)^{-\frac{p}{2}} \end{aligned}$$

15 $$\begin{aligned} \Lambda _i&= (z_1+\grave{x}_0+\mu -\delta _i^2 )z_2+(z_3+\grave{y}_0)z_4 \nonumber \\&= \mathscr {K}_{i,1}z_2+\mathscr {K}_{1,3}z_4 \end{aligned}$$

where $$j=\{1,3\},\; i=\{1,2,3\},\; p\in N^+ \text {and} \;\delta _i^j= {\left\{ \begin{array}{ll} 1 & \text {if} \qquad i=j \\ 0 & \text {if} \qquad i\ne j \end{array}\right. }$$.

The Lie operator, obtained by substituting eqs. (10) to (14) into eq. (4), can be expressed as:

16 $$\begin{aligned} D&=\sum \limits _{i =1}^{4}\frac{dz_i}{dt}\frac{\partial }{\partial z_i} \nonumber \\&= z_2\frac{\partial }{\partial z_1} + \left( 2z_4 +\left[ \mathscr {K}_{3,1}- \frac{1}{n^2}\left\{ (1-\mu )\mathscr {K}_{1,1}\varrho _1^3 +\frac{3J_{2,J}R^{2}_{J}(1-\mu )}{2}\mathscr {K}_{1,1}\varrho _1^5 \right. \right. \right. \nonumber \\&\left. \left. \left. +\mu \mathscr {K}_{2,1}\varrho _2^3 +\frac{3\mu R^{2}_{E}}{2 }(J_{2,E}-14C_{22,E})\mathscr {K}_{2,1}\varrho _2^5+ 30\mu C_{22,E}R^{2}_{E}\mathfrak {L}_{2,1}^{3}\varrho _2^7 \right\} \right] \right) \frac{\partial }{\partial z_2} +z_4\frac{\partial }{\partial z_3}\nonumber \\&+ \left( -2z_2 + \left[ \mathscr {K}_{1,3}- \frac{1}{n^2}\left\{ (1-\mu )\mathscr {K}_{1,3}\varrho _1^3 +\frac{3J_{2,J}R^{2}_{J}(1-\mu )}{2}\mathscr {K}_{1,3}\varrho _1^5 +\mu \mathscr {K}_{1,3}\varrho _2^3\right. \right. \right. \nonumber \\&\left. \left. \left. +\frac{3\mu R^{2}_{E}}{2 }(J_{2,E}+14C_{22,E})\mathscr {K}_{1,3}\varrho _2^5- 30\mu C_{22,E}R^{2}_{E}\mathfrak {L}_{1,3}^{3}\varrho _2^7\right\} \right] \right) \frac{\partial }{\partial z_4} \end{aligned}$$

Using Equations (8), (9) and (16), the solutions to Equations (10) can be obtained through a series expansion:

17 $$\begin{aligned} z_i(\tau )=e^{\tau D}\ z_i(0)=\sum \limits _{n=0}^{\infty }\left( \frac{\tau ^{n}D^{n}}{n!}\right) \ z_i(0), \end{aligned}$$

Here, $\tau$ represents the current step size, which is defined as follows:

$$\begin{aligned} \tau =t_{j}-t_{j-1} \end{aligned}$$

To enable effective applications of the method, a recurrence relation must be derived to determine any order of the Lie operator. For $z_1$ and by applying operator *D* and its power we get

18 $$\begin{aligned} D^0z_1=z_1,\quad Dz_1=z_2,\;\text {and}\; D^nz_1=D^{(n-1)}z_2 \end{aligned}$$

and for $$z_3$$

19 $$\begin{aligned} D^0z_3=z_4,\quad Dz_3=z_4,\;\text {and}\; D^nz_3=D^{(n-1)}z_4 \end{aligned}$$

Now for $z_2$ and $z_4$, we must get the *D* operator and its power for $\mathscr {K}_{i,j}$, $\mathfrak {L}_{i,j}^{p}$, $\varrho _i^p$, and $\Lambda _i$

20 $$\begin{aligned} D^0\mathscr {K}_{i,j}=\mathscr {K}_{i,j},\quad D\mathscr {K}_{i,j}=z_j,\;\text {and}\; D^n\mathscr {K}_{i,j}=D^{(n-1)}z_j \end{aligned}$$

21 $$\begin{aligned} D^0\mathfrak {L}_{i,j}^{p}=\mathfrak {L}_{i,j}^{p},\quad D\mathfrak {L}_{i,j}^{p}=p\mathfrak {L}_{i,j}^{p-1}Dz_j \quad \text {and} \quad D^n\mathfrak {L}_{i,j}^{p}=p\sum \limits _{l=0}^{n-1}\left( {\begin{array}{c}n-1\\ l\end{array}}\right) \left( D^{n-l-1}\mathfrak {L}_{i,j}^{p-1}D^{l+1}z_{j}\right) \end{aligned}$$

22 $$\begin{aligned} D^0\Lambda _i=\Lambda _i,\quad \text {and} \quad D^n\Lambda _i=\sum \limits _{l=0}^{n}\left( {\begin{array}{c}n\\ l\end{array}}\right) \left( D^{n-l}\mathscr {K}_{i,1}D^lz_{2}+D^{n-l}\mathscr {K}_{1,3}D^lz_{4}\right) \end{aligned}$$

From^25^, we have

23 $$\begin{aligned} D^{n}\varrho _i^p=\varrho _i^2\sum \limits _{k=0}^{n-1}a_{n,k+1}D^{n-1-k}\varrho _i^pD^{k}\Lambda _i. \end{aligned}$$

The coefficients $a_{i,j}$ have the forms for $n\ge 1:$

24 $$\begin{aligned} {\left\{ \begin{array}{ll} a_{n,n}=-p \\ a_{n,k}=a_{n-1,k-1}+a_{n-1,k}\text { for }k\ge 1 \\ a_{n,1}=a_{n-1,1}-2 \end{array}\right. } \end{aligned}$$

For $$z_2$$

25 $$\begin{aligned} D^nz_2=&D^{(n-1)}Dz_2 \nonumber \\ =&\left( 2D^{(n-1)}z_4 + \left[ D^{(n-1)}\mathscr {K}_{3,1} \right. \right. \nonumber \\&- \frac{1}{n^2}\left\{ (1-\mu ) \sum \limits _{l=0}^{n-1}\left( {\begin{array}{c}n-1\\ l\end{array}}\right) D^{n-l-1}\mathscr {K}_{1,1}D^l\varrho _1^3 \right. \nonumber \\&+\frac{3J_{2,J}R^{2}_{J}(1-\mu )}{2}\sum \limits _{l=0}^{n-1}\left( {\begin{array}{c}n-1\\ l\end{array}}\right) D^{n-l-1}\mathscr {K}_{1,1}D^l\varrho _1^5 \nonumber \\&+\mu \sum \limits _{l=0}^{n-1}\left( {\begin{array}{c}n-1\\ l\end{array}}\right) D^{n-l-1}\mathscr {K}_{2,1}D^l\varrho _2^3 \nonumber \\&+\frac{3\mu R^{2}_{E}}{2}(J_{2,E}-14C_{22,E})\sum \limits _{l=0}^{n-1}\left( {\begin{array}{c}n-1\\ l\end{array}}\right) D^{n-l-1}\mathscr {K}_{2,1}D^l\varrho _2^5 \nonumber \\&\left. \left. \left. +30\mu C_{22,E}R^{2}_{E}\sum \limits _{l=0}^{n-1}\left( {\begin{array}{c}n-1\\ l\end{array}}\right) D^{n-l-1}\mathfrak {L}_{2,1}^3D^l\varrho _2^7\right\} \right] \right) \end{aligned}$$

Using equations (22), (23), and (24) into eqaution (25), we get

26 $$\begin{aligned}&D^nz_2=2D^{(n-1)}z_4 + \left[ D^{(n-1)}\mathscr {K}_{3,1} \right. \nonumber \\&- \frac{1}{n^2}\left\{ (1-\mu )\varrho _1^2 \sum \limits _{l=0}^{n-1}\sum \limits _{k=0}^{l-1}\sum \limits _{j=0}^{k}a_{l,k+1}\left( {\begin{array}{c}n-1\\ l\end{array}}\right) \left( {\begin{array}{c}k\\ j\end{array}}\right) D^{n-l-1}\mathscr {K}_{1,1}\right. \nonumber \\&\qquad \qquad \qquad \qquad \qquad \qquad (D^{k-j}\mathscr {K}_{1,1}D^jz_{2}+D^{k-j}\mathscr {K}_{1,3}D^jz_{4}) D^{l-1-k}\left( \varrho _1^3+\frac{3J_{2,J}R^{2}_{J}}{2} \varrho _1^5\right) \nonumber \\&\qquad \qquad +\mu \varrho _2^2 \sum \limits _{l=0}^{n-1}\sum \limits _{k=0}^{l-1}\sum \limits _{j=0}^{k}a_{l,k+1}\left( {\begin{array}{c}n-1\\ l\end{array}}\right) \left( {\begin{array}{c}k\\ j\end{array}}\right) D^{n-l-1}\mathscr {K}_{2,1}(D^{k-j}\mathscr {K}_{2,1}D^jz_{2}\nonumber \\&\qquad \qquad \qquad \qquad \qquad \qquad +D^{k-j}\mathscr {K}_{1,3}D^jz_{4}) D^{l-1-k}\left( \varrho _2^3+\frac{3R^{2}_{E}}{2}(J_{2,E}-14C_{22,E}) \varrho _2^5\right) \nonumber \\&\qquad \qquad +90\mu C_{22,E}R^{2}_{E} \varrho _2^2 \sum \limits _{l=0}^{n-1}\;\sum \limits _{h=0}^{n-l-2}\; \sum \limits _{k=0}^{l-1}\sum \limits _{j=0}^{k}a_{l,k+1}\left( {\begin{array}{c}n-1\\ l\end{array}}\right) \left( {\begin{array}{c}n-l-2\\ h\end{array}}\right) \left( {\begin{array}{c}k\\ j\end{array}}\right) D^{n-l-h-2}\mathfrak {L}_{2,1}^2\nonumber \\&\qquad \qquad \qquad \qquad \qquad \qquad \left. \left. D^{h+1}z_1(D^{k-j}\mathscr {K}_{2,1}D^jz_{2}+D^{k-j}\mathscr {K}_{1,3}D^jz_{4}) D^{l-1-k} \varrho _2^7 \right\} \right] \end{aligned}$$

Similarly for $$z_4$$

27 $$\begin{aligned}&D^nz_4=-2D^{(n-1)}z_2 + \left[ D^{(n-1)}\mathscr {K}_{1,3} \right. \nonumber \\&- \frac{1}{n^2}\left\{ (1-\mu )\varrho _1^2 \sum \limits _{l=0}^{n-1}\sum \limits _{k=0}^{l-1}\sum \limits _{j=0}^{k}a_{l,k+1}\left( {\begin{array}{c}n-1\\ l\end{array}}\right) \left( {\begin{array}{c}k\\ j\end{array}}\right) D^{n-l-1}\mathscr {K}_{1,3}\right. \nonumber \\&\qquad \qquad \qquad \qquad \qquad \qquad (D^{k-j}\mathscr {K}_{1,1}D^jz_{2}+D^{k-j}\mathscr {K}_{1,3}D^jz_{4}) D^{l-1-k}\left( \varrho _1^3+\frac{3J_{2,J}R^{2}_{J}}{2} \varrho _1^5\right) \nonumber \\&\qquad \qquad +\mu \varrho _2^2 \sum \limits _{l=0}^{n-1}\sum \limits _{k=0}^{l-1}\sum \limits _{j=0}^{k}a_{l,k+1}\left( {\begin{array}{c}n-1\\ l\end{array}}\right) \left( {\begin{array}{c}k\\ j\end{array}}\right) D^{n-l-1}\mathscr {K}_{1,3}(D^{k-j}\mathscr {K}_{2,1}D^jz_{2}\nonumber \\&\qquad \qquad \qquad \qquad \qquad \qquad +D^{k-j}\mathscr {K}_{1,3}D^jz_{4}) D^{l-1-k}\left( \varrho _2^3+\frac{3R^{2}_{E}}{2}(J_{2,E}+14C_{22,E}) \varrho _2^5\right) \nonumber \\&\qquad \qquad -90\mu C_{22,E}R^{2}_{E} \varrho _2^2 \sum \limits _{l=0}^{n-1}\;\sum \limits _{h=0}^{n-l-2}\; \sum \limits _{k=0}^{l-1}\sum \limits _{j=0}^{k}a_{l,k+1}\left( {\begin{array}{c}n-1\\ l\end{array}}\right) \left( {\begin{array}{c}n-l-2\\ h\end{array}}\right) \left( {\begin{array}{c}k\\ j\end{array}}\right) D^{n-l-h-2}\mathfrak {L}_{1,3}^2\nonumber \\&\qquad \qquad \qquad \qquad \qquad \qquad \left. \left. D^{h+1}z_3(D^{k-j}\mathscr {K}_{2,1}D^jz_{2}+D^{k-j}\mathscr {K}_{1,3}D^jz_{4}) D^{l-1-k} \varrho _2^7 \right\} \right] \end{aligned}$$

Hence the solution of the system will be

28 $$\begin{aligned} z_1=&z_1(0)+\sum _{n=1}^{\infty }\frac{\tau ^n}{n!}D^{(n-1)}z_2(0) \end{aligned}$$

29 $$\begin{aligned} z_2=&z_2(0) +2\sum _{n=1}^{\infty }\frac{\tau ^n}{n!}D^{(n-1)}z_4(0) + \left[ \sum _{n=1}^{\infty }\frac{\tau ^n}{n!}D^{(n-1)}\mathscr {K}_{3,1}(0) \right. \nonumber \\&- \frac{1}{n^2}\left\{ (1-\mu )\varrho _1^2(0) \sum _{n=1}^{\infty }\sum \limits _{l=0}^{n-1}\sum \limits _{k=0}^{l-1}\sum \limits _{j=0}^{k}\frac{\tau ^n}{n!}a_{l,k+1}\left( {\begin{array}{c}n-1\\ l\end{array}}\right) \left( {\begin{array}{c}k\\ j\end{array}}\right) D^{n-l-1}\mathscr {K}_{1,1}(0)\right. \nonumber \\&\qquad \qquad \qquad \qquad \qquad \qquad (D^{k-j}\mathscr {K}_{1,1}(0)D^jz_{2}(0)+D^{k-j}\mathscr {K}_{1,3}(0)D^jz_{4}(0)) D^{l-1-k}\left( \varrho _1^3+\frac{3J_{2,J}R^{2}_{J}}{2} \varrho _1^5\right) \Bigg |_{\textbf{z}(0)} \nonumber \\&\qquad \qquad +\mu \varrho _2^2(0) \sum _{n=1}^{\infty }\sum \limits _{l=0}^{n-1}\sum \limits _{k=0}^{l-1}\sum \limits _{j=0}^{k}\frac{\tau ^n}{n!}a_{l,k+1}\left( {\begin{array}{c}n-1\\ l\end{array}}\right) \left( {\begin{array}{c}k\\ j\end{array}}\right) D^{n-l-1}\mathscr {K}_{2,1}(0)(D^{k-j}\mathscr {K}_{2,1}(0)D^jz_{2}(0)\nonumber \\&\qquad \qquad \qquad \qquad \qquad \qquad +D^{k-j}\mathscr {K}_{1,3}(0)D^jz_{4}(0)) D^{l-1-k}\left( \varrho _2^3+\frac{3R^{2}_{E}}{2}(J_{2,E}-14C_{22,E}) \varrho _2^5\right) \Bigg |_{\textbf{z}(0)} \nonumber \\&\qquad \qquad +90\mu C_{22,E}R^{2}_{E} \varrho _2^2(0) \sum _{n=1}^{\infty }\sum \limits _{l=0}^{n-1}\;\sum \limits _{h=0}^{n-l-2}\; \sum \limits _{k=0}^{l-1}\sum \limits _{j=0}^{k}\frac{\tau ^n}{n!}a_{l,k+1}\left( {\begin{array}{c}n-1\\ l\end{array}}\right) \left( {\begin{array}{c}n-l-2\\ h\end{array}}\right) \left( {\begin{array}{c}k\\ j\end{array}}\right) D^{n-l-h-2}\mathfrak {L}_{2,1}^2(0)\nonumber \\&\qquad \qquad \qquad \qquad \qquad \qquad \left. \left. D^{h+1}z_1(0)(D^{k-j}\mathscr {K}_{2,1}(0)D^jz_{2}(0)+D^{k-j}\mathscr {K}_{1,3}(0)D^jz_{4}(0)) D^{l-1-k} \varrho _2^7(0) \right\} \right] \end{aligned}$$

30 $$\begin{aligned} z_3=&z_3(0)+\sum _{n=1}^{\infty }\frac{\tau ^n}{n!}D^{(n-1)}z_4(0)\end{aligned}$$

31 $$\begin{aligned} z_4=&z_2(0) -2\sum _{n=1}^{\infty }\frac{\tau ^n}{n!}D^{(n-1)}z_2(0) + \left[ \sum _{n=1}^{\infty }\frac{\tau ^n}{n!}D^{(n-1)}\mathscr {K}_{1,3}(0) \right. \nonumber \\&- \frac{1}{n^2}\left\{ (1-\mu )\varrho _1^2(0) \sum _{n=1}^{\infty }\sum \limits _{l=0}^{n-1}\sum \limits _{k=0}^{l-1}\sum \limits _{j=0}^{k}\frac{\tau ^n}{n!}a_{l,k+1}\left( {\begin{array}{c}n-1\\ l\end{array}}\right) \left( {\begin{array}{c}k\\ j\end{array}}\right) D^{n-l-1}\mathscr {K}_{1,3}(0)\right. \nonumber \\&\qquad \qquad \qquad \qquad \qquad \qquad (D^{k-j}\mathscr {K}_{1,1}(0)D^jz_{2}(0)+D^{k-j}\mathscr {K}_{1,3}(0)D^jz_{4}(0)) D^{l-1-k}\left( \varrho _1^3+\frac{3J_{2,J}R^{2}_{J}}{2} \varrho _1^5\right) \Bigg |_{\textbf{z}(0)} \nonumber \\&\qquad \qquad +\mu \varrho _2^2(0) \sum _{n=1}^{\infty }\sum \limits _{l=0}^{n-1}\sum \limits _{k=0}^{l-1}\sum \limits _{j=0}^{k}\frac{\tau ^n}{n!}a_{l,k+1}\left( {\begin{array}{c}n-1\\ l\end{array}}\right) \left( {\begin{array}{c}k\\ j\end{array}}\right) D^{n-l-1}\mathscr {K}_{1,3}(0)(D^{k-j}\mathscr {K}_{2,1}(0)D^jz_{2}(0)\nonumber \\&\qquad \qquad \qquad \qquad \qquad \qquad +D^{k-j}\mathscr {K}_{1,3}(0)D^jz_{4}(0)) D^{l-1-k}\left( \varrho _2^3+\frac{3R^{2}_{E}}{2}(J_{2,E}+14C_{22,E}) \varrho _2^5\right) \Bigg |_{\textbf{z}(0)} \nonumber \\&\qquad \qquad -90\mu C_{22,E}R^{2}_{E} \varrho _2^2(0) \sum _{n=1}^{\infty }\sum \limits _{l=0}^{n-1}\;\sum \limits _{h=0}^{n-l-2}\; \sum \limits _{k=0}^{l-1}\sum \limits _{j=0}^{k}\frac{\tau ^n}{n!}a_{l,k+1}\left( {\begin{array}{c}n-1\\ l\end{array}}\right) \left( {\begin{array}{c}n-l-2\\ h\end{array}}\right) \left( {\begin{array}{c}k\\ j\end{array}}\right) D^{n-l-h-2}\mathfrak {L}_{1,3}^2(0)\nonumber \\&\qquad \qquad \qquad \qquad \qquad \qquad \left. \left. D^{h+1}z_3(0)(D^{k-j}\mathscr {K}_{2,1}(0)D^jz_{2}(0)+D^{k-j}\mathscr {K}_{1,3}(0)D^jz_{4}(0)) D^{l-1-k} \varrho _2^7(0) \right\} \right] \end{aligned}$$

Equations (28)-(31) represent an approximate analytical solution for the equations of motion of the system. This solution captures the dynamical behavior of a spacecraft near the triangular libration points. The solution not only characterizes families of periodic orbits around the triangular points but also serves as an effective tool for validating and complementing numerical simulations. The Lie–series method is implemented with a constant time step, with expansions carried out up to the eighth order, as this level of truncation already ensures highly accurate solutions. In fact, the difference between the eighth-order and ninth-order solutions was on the order of $10^{-15}$. In parallel, direct numerical integrations are performed using the Sundials CVODE solver, which employs an adaptive step-size scheme.

## Classical vs. full perturbation models: tadpole and horseshoe Orbits

**Fig. 1:**
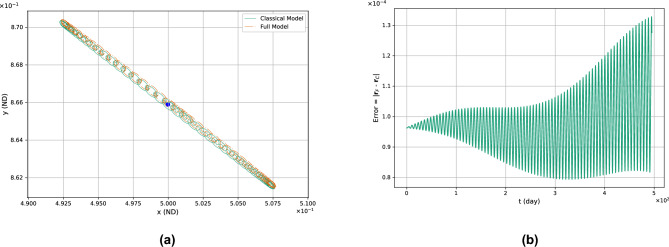
(**a**) compares the tadpole orbit obtained under the classical circular restricted three-body problem and the perturbed model for $\beta$ = 0.00001. (**b**) shows the time evolution of the state difference between the two models. The units used in the plot are non-dimensional (ND), and the blue sphere represents the triangular point $L_4$. The simulations are performed with initial conditions $x_0 = 0.4999797,\; x'_0 = 0.0001,\; y_0 = 0.8660341,\; \text {and}\; y'_0 = 0.0001$.

**Fig. 2:**
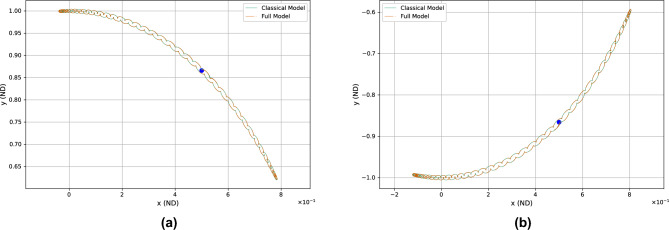
(**a**) and (**b**) compare the tadpole orbits around the triangular points $L_{4}$ and $L_{5}$, respectively, under both the classical circular restricted three-body problem and the perturbed model for $\beta = 0.001$. The units used in the plot are non-dimensional (ND), and the blue sphere represents the triangular point $L_5$. The simulations are performed with initial conditions $x_0 = 0.5004747,\; x'_0 = 0.0001,\; y_0 = 0.8668914 (L_4),\; \text {or}\; -0.8668914 (L_5),\; \text {and}\; y'_0 = 0.0001$.

**Fig. 3:**
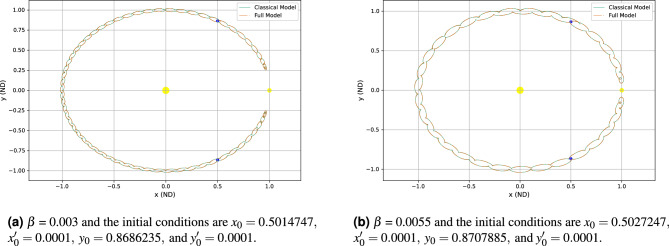
(**a**) and (**b**) compare the horseshoe orbits around the triangular points $L_{4}$, under both the classical circular restricted three-body problem and the perturbed model with different initial conditions. The units in the plot are non-dimensional (ND). The large and small yellow spheres represent Jupiter and Europa, respectively.

In the restricted three-body problem, there exist different types of orbits close to the equilateral points $L_4, L_5$. This motion is valid only for a small magnitude displacement from the equilibrium point. The most significant orbits around the equilateral equilibrium points are the tadpole and horseshoe orbits. To achieve a proper graphical and numerical representation of periodic orbits in the restricted three-body problem, it is essential to integrate the governing equations of motion. This process requires utilizing a set of initial conditions. The periodicity of the orbit requires that the initial state vector be periodic. When this condition is not fulfilled, the initial state can be refined using differential correction methods^16^^,^^27^. It is usually difficult to determine initial conditions that lead to a periodic orbit, unless the correct numerical methodology is applied. In the present work, to identify the appropriate initial conditions, we follow the method used in Taylor^17^. Assuming that the initial condition can generate a periodic orbit, the equations of motion can be numerically integrated using the CVODE integrator, which is a solver for stiff and non-stiff ordinary differential equations of the form $y^{\prime }=f(y, t)$^28^. The software package CVODE utilizes flexible numerical techniques that adjust both the order of accuracy and the step size. For nonstiff systems, it employs the Adams-Moulton formulas with orders ranging from 1 to 12, while for stiff systems, it uses the Backward Differentiation Formulas with orders from 1 to 5.

Following Taylor^17^, we assume that the spacecraft *P*(*x*, *y*) is located along the line connecting Jupiter with the triangular point $L_4$ or $L_5$. This line represents the closest approach of the spacecraft to the point. A suitable approximation for a periodic orbit about the point is that the velocity magnitude of the spacecraft *V* varies only slowly, such that $\frac{\partial V}{\partial t}$ and $\frac{\partial V^2}{\partial t^2}$ tend to zero. Let us identify $\beta$ with the distance between the spacecraft and the libration center $L_4$ or $L_5$, measured along the straight line connecting Jupiter with the triangular point $L_4$. Then the starting value $d_0$, representing the spacecraft’s distance from Jupiter, is given by $d_0=1+\beta$. In the synodic-rotating frame, the coordinates of the spacecraft can then be expressed as $x=\frac{(1+\beta )}{2}-\mu$ and $y=(1+\beta ) \frac{\sqrt{3}}{2}$. By varying the value of $\beta$, we can obtain several initial conditions that characterize the dynamical behavior of the spacecraft close to the point $L_4$.

In the current analysis, we consider two distinct dynamical models. The first case corresponds to the classical CR3BP formulation (green curve), whereas the second accounts for the perturbative effects arising from the oblateness of Jupiter and Europa together with Europa’s equatorial ellipticity (orange curve). We examine their respective roles in shaping tadpole and horseshoe orbits around the triangular equilibrium points. In Fig. 1a, the left panel illustrates the trajectory of a tadpole orbit around the triangular equilibrium point $L_4$ in the Jupiter–Europa system, computed under two dynamical frameworks: the classical restricted three-body problem and the perturbed model. We observe that, for $\beta =0.00001$, the inclusion of Jupiter’s and Europa’s oblateness, together with Europa’s equatorial ellipticity, introduces subtle perturbations that gradually distort and shift the tadpole orbit. Although the perturbations are small in magnitude relative to the classical case, their cumulative influence alters the geometry of the tadpole orbit. This demonstrates that even minor physical corrections can significantly affect long-period dynamics in the system. In the right panel, Fig. 1b quantifies the divergence between the two models by plotting the state difference at each time step. Over long-period evolution, the deviations between the classical and augmented models accumulate, resulting in measurable differences in the orbital state. Fig.2a illustrates the tadpole orbit around $L_4$, while Fig.2b shows the corresponding orbit around $L_5$, both computed under identical initial conditions with $\beta =0.001$. In each case, the classical model yields a smooth, symmetric trajectory, whereas the full model introduces subtle deviations and a slightly different orbital period. Figs.3a and b present a comparison between the classical and full models. The comparison is conducted to examine the influence of the classical and full models on horseshoe orbits, using different initial conditions in each case. The two figures highlight how the classical model yields smooth, symmetric trajectories, whereas the full model induces a slight shift in the orbit and a small variation in the period. Although the two dynamical models show no major differences, the subtle cumulative effects of perturbations must be accounted for in long-term studies to achieve the high-precision results required for space mission design.

## Comparison of Lie series and numerical integration approaches

**Fig. 4:**
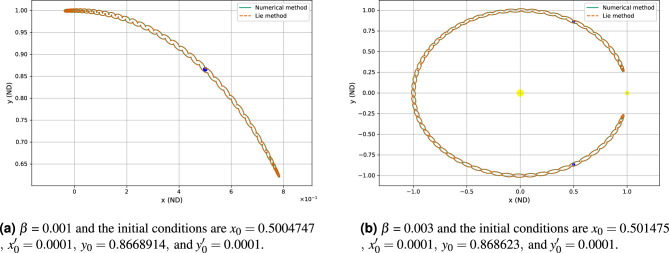
(**a)** (left) compares tadpole orbits, and (**b**) (right) compares horseshoe orbits, generated with the Lie-series method and numerical integration under the full perturbation model, using different initial conditions. The units in the plot are non-dimensional (ND).

**Fig. 5:**
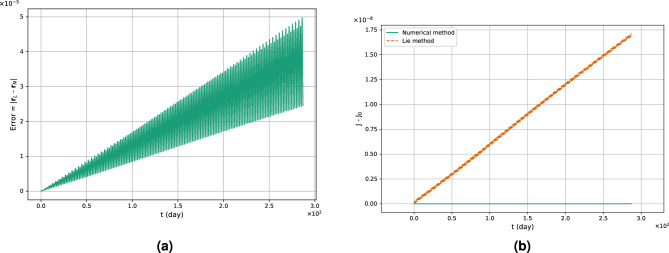
The left panel shows the time evolution of the state difference between numerical integration and the Lie-series solution for $\beta = 0.001$, while the right panel compares the Jacobi constant from both methods. The initial conditions are $x_0 = 0.5004747,\; x'_0 = 0.0001,\; y_0 = 0.8668914,\; \text {and}\; y'_0 = 0.0001$.

This section is devoted to examining the degree of agreement between the analytical solution obtained through the Lie-series method and the numerical integration approach in the perturbed dynamical model. Figs.4a and b compare the analytical solution obtained via the Lie series method (orange dashed curve) with the numerical integration (green solid curve) for the perturbed model with $\beta =0.001$ and $\beta =0.003$, respectively. Together, the two figures highlight that the Lie series analytical method yields results that are highly consistent with the numerical integration for both tadpole and horseshoe orbits in the perturbed system. Fig.5a shows the time evolution of the state difference between the numerical integration and the Lie series analytical solution with $\beta =0.001$. The error, defined as $\left| r_{n} - r_{l} \right|$, grows gradually with oscillatory variations, reaching the order of $10^{-5}$ over the integration span of about 300 days. In addition, Fig.5b compares the conservation of the Jacobi constant between the two methods. Together, the figures demonstrate that the Lie series analytical solution agrees well with the numerical integration, with only small state differences and a modest drift in the Jacobi constant.

Now, we examine the linear stability of the Jupiter–Europa system under the influence of perturbing forces arising from the oblateness of both Jupiter and its satellite Europa, together with the equatorial ellipticity of Europa. To evaluate the stability characteristics, the eigenvalues of the characteristic equation are determined. This approach facilitates the identification of stability domains and the onset of dynamical instabilities. Let the coordinates of the equilibrium point be denoted as $(x_0, y_0)$. Assume $\alpha$ and $\gamma$ denote small displacements of the third body from the point $(x_0, y_0)$, then the location of the third body at any time is given as $x = x_0 + \alpha , \quad y = y_0 + \gamma$. Assuming that $\alpha$ and $\gamma$ are sufficiently small, the Taylor expansions of $U_x$ and $U_y$ about the equilibrium point $(x_0, y_0)$ up to linear order terms in $\alpha , \gamma$ can be written as

32 $$\begin{aligned} U_x = \alpha U_{xx} + \gamma U_{xy} \qquad U_y = \alpha U_{yx} + \gamma U_{yy} \end{aligned}$$

Therefore, the equations of motion in the neighborhood of $(x_0, y_0)$ can be written as

33 $$\begin{aligned} \ddot{\alpha } - 2n \dot{\gamma }&= \alpha U_{xx} + \gamma U_{xy}, \end{aligned}$$

34 $$\begin{aligned} \ddot{\gamma } + 2n \dot{\alpha }&= \alpha U_{yx} + \gamma U_{yy}. \end{aligned}$$

To investigate the motion of the infinitesimal body around the equilibrium point, let us suppose $\alpha = A e^{\lambda t}, \quad \gamma = B e^{\lambda t}$. Where $A, B, \lambda$ are parameters to be determined. Substituting $\alpha$ and $\gamma$ into equations (33)–(34) yields the characteristic equation: $\lambda ^4 + P_1 \lambda ^2 + Q_1 = 0$, where $P_1 = 4n^2 - U_{xx} - U_{yy}$ and $Q_1 = U_{xx} U_{yy} - (U_{xy})^2$. The characteristic roots of this equation are given as

$$\lambda = \pm \frac{1}{2}\sqrt{-P_1 \pm \sqrt{P_1^2 - 4Q_1}}.$$

For the linear stability of equilibrium points, the character of the roots is determined by the sign of the discriminant $P_1^2 - 4Q_1$. When the discriminant satisfies $P_1^2 - 4Q_1> 0$, the resulting roots are purely imaginary, and the motion is linearly stable. Setting the discriminant equal to zero defines the critical mass ratio.

Using the above method, we determined the critical mass ratio of the system and found its value to be $\mu _c=0.024988279$ when all perturbing forces are taken into account. Beyond this threshold, the system transitions into dynamical instability. By comparing this value with the classical critical mass ratio, we find that it is smaller than the classical value. Consequently, the regions of stability are reduced relative to the classical case, and the system undergoes instability earlier than in the classical case. Beyond the critical mass ratio, the tadpole and horseshoe orbits transition to chaotic trajectories, indicating the onset of dynamical instability in the system. The determination of stability in the circular restricted three-body problem depends primarily on the reduced mass parameter. However, when additional perturbative forces such as the oblateness of the primaries and the equatorial ellipticity are present, they also play a role in shaping the stability regions. Nevertheless, the dominant influence remains that of the reduced mass. Our results are close to those obtained by Yousuf et al.^23^, with slight differences attributable to the adoption of different dynamical models. Europa’s ellipticity should be taken into account in long-term dynamical studies to ensure high accuracy.

## Surfaces of zero velocity

In space missions, it is necessary to identify the dynamical transport corridor between the primaries of the dynamical systems. The Jacobi constant is usually used to identify these gateways. It defines the zero-velocity surfaces, which act as boundaries between forbidden and allowed regions of motion. For a given mass parameter $\mu$, each equilibrium point has a specific Jacobi constant value, $CL_i, i=1, \dots , 5$. The correct ordering of Jacobi constants for the libration points is $CL_1> CL_2> CL_3> CL_4 \approx CL_5$. The conduit can only exist when the value of the Jacobi constant lies below the $L_1$threshold. Therefore, the opening condition is $C \le CL_1$. If this condition is not verified, then $(2 \Omega -C)<0$, the gateways between the primaries or between them and Lagrange points become locked. Tables 1 and 2 summarize the comparison between the classical circular restricted three-body problem and the perturbed model, specifically highlighting the differences in the locations of the libration points and the corresponding values of the Jacobi constant.

**Fig. 6:**
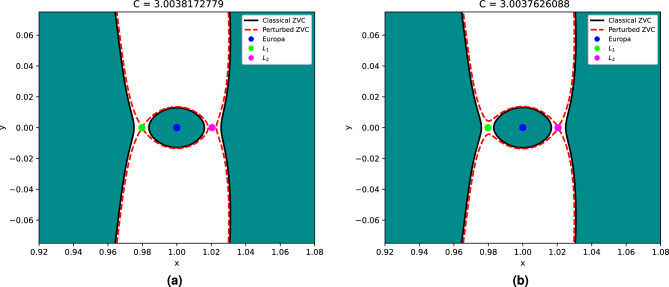
The Jacobi constant values at which the necks around the $L_1$ and $L_2$ points open in the perturbed case (red dashed line). The left panel shows the Jacobi constant at which the neck around $L_1$ opens, while the right shows the corresponding value for $L_2$. The units used in the plot are non-dimensional (ND).

**Fig. 7:**
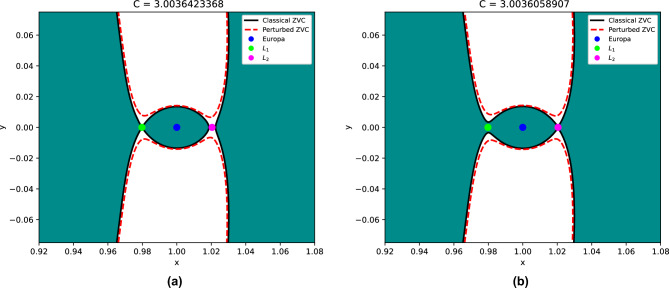
The Jacobi constant values at which the necks around the $L_1$ and $L_2$ points open in the classical case (black line). The left panel shows the Jacobi constant at which the neck around $L_1$ opens, while the right shows the corresponding value for $L_2$. The units used in the plot are non-dimensional (ND).

Figs. 6a, b, 7a, and b illustrate the zero-velocity curves (ZVCs) in the Jupiter–Europa restricted three-body system, comparing the classical model (black line) with the perturbed one (red dashed line). The figures compare the Jacobi constant values at which the necks around the points $L_1$ and $L_2$ are open, permitting a small particle to transfer between Jupiter and Europa or to escape. The plots depict the spacecraft’s ability to transit through the Hill’s neck as the Jacobi constant decreases. To visualize clearly the opening and closing of the gateway between the Jupiter and Europa domains, we zoom in on the region near Europa. These surfaces are plotted in the range $[CL_1 - \epsilon , \; CL_1 + \epsilon ]$, where $\epsilon$ is a small quantity of order $\approx O(10^{-5})$. The inclusion of the higher-order gravity terms alters the gravitational field within the Jupiter-Europa system. Thus, the modified effective potential reshapes the boundaries of the Hill regions. When modeling both Jupiter and Europa as perfect spherical bodies (the classical case), we obtain the classical closure value $CL_1 \approx 3.003642790166$. Once the oblateness of Jupiter and Europa’s non-spherical mass distribution are introduced, an increase in the Jacobi constant exists. So the condition for disconnection of the two regions, around Jupiter and Europa, is now verified at $CL_1 \approx 3.003820225122$ rather than 3.003642790166 (see Table 2). After this closure value, the zero-velocity surfaces remain connected, and the neck is closed. The spacecraft does not have enough energy to transfer between Europa and Jupiter or reach the equilibrium points. Furthermore, in the general planar three-body problem investigated by Bozis^29^, surfaces of zero velocity were found that extend to infinity and others that are bounded. However, in all cases, unlike the circular restricted three-body problem, no closed surface was found to ensure bounded motion. Instead, they reveal possible escape routes, forbidden regions, and the dependence of motion on energy and angular momentum.

**Table 1:** Comparison of Lagrange point coordinates in the classical and perturbed models.

Point	Classical (CR3BP)		Perturbed model	
	*x*	*y*	*x*	*y*
$L_1$	0.979764104589	0.000000000000	0.979793235154	0.000000000000
$L_2$	1.020461385982	0.000000000000	1.020487944356	0.000000000000
$L_3$	−1.000010533406	0.000000000000	−1.000093955423	0.000000000000
$L_4$	0.499974719825	0.866025403784	0.500058145929	0.866073561983
$L_5$	0.499974719824	−0.866025403785	0.500058145928	−0.866073561983

**Table 2:** Comparison of Jacobi Constant values $C_{L_i}$ in the classical and perturbed models.

Point	Classical	Perturbed	$\Delta C$ (Shift)
$L_1$	3.003642790166	3.003820225122	+0.000177434956
$L_2$	3.003609081999	3.003766126360	+0.000157044361
$L_3$	3.000025280162	3.000192145072	+0.000166864910
$L_4$	2.999974720464	3.000141577992	+0.000166857528
$L_5$	2.999974720464	3.000141577992	+0.000166857528

## Conclusion

Designing spaceflights necessitates in-depth and comprehensive studies to ensure mission success. This involves studying the motion near planetary satellites and determining periodic orbits around the equilibrium points of dynamic systems. In this work, we studied the Jupiter-Europa system, considering perturbations induced by the oblateness of both Jupiter and Europa, as well as Europa’s equatorial ellipticity. Under the perturbations considered, and for different initial conditions, we determined tadpole and horseshoe orbits in the Jupiter-Europa system. It is observed that varying the initial conditions results in orbits of different sizes and periods. We investigated the tadpole and horseshoe orbits under two different models—the classical circular restricted three-body problem and the perturbed model—and found that the differences between them are relatively small. Nevertheless, these subtle perturbations should be accounted for to ensure high efficiency in mission design, particularly for long-period space missions. In addition, the results confirmed that the system is sensitive to the initial conditions. Furthermore, the stability analysis shows that the Jupiter–Europa system remains linearly stable under the considered perturbations within the range $0<\mu \le \mu _{c}$, beyond which the system transitions to instability.

In the perturbed circular restricted three-body problem, the accessibility of equilibrium points depends on the value of the Jacobi constant. If its value is fixed, certain regions of motion are blocked by zero-velocity surfaces. To allow a spacecraft to reach the vicinity of an equilibrium point, the Jacobi constant must vary so that the zero-velocity surfaces open up and make those regions dynamically accessible. Considering the collinear point $L_1$, the value of the Jacobi constant is $CL_1= 3.003820225122$. Under this condition, a small body orbiting Jupiter would be unable to reach Europa, and vice versa. To connect the internal regions, we slightly reduce the value of the Jacobi constant and set $C \approx 3.0038172779$. At this value, the two regions do indeed connect, and a bottleneck appears around $L_1$; Europa is accessible. A further reduction of the Jacobi constant extends the region around the smaller primary to the point $L_2$. Also, for the small body to reach $L_3$, its energy must increase significantly. Furthermore, the Jacobi constant value $CL_4= 3.000141577992$ opens the entire synodic plane to the small body. The current findings could facilitate further exploration of the generalized restricted three-body problem in space operations.

## Data Availability

All data generated or analyzed during this study are included in this published article.
